# Aging and Network Properties: Stability Over Time and Links with Learning during Working Memory Training

**DOI:** 10.3389/fnagi.2017.00419

**Published:** 2018-01-04

**Authors:** Alexandru D. Iordan, Katherine A. Cooke, Kyle D. Moored, Benjamin Katz, Martin Buschkuehl, Susanne M. Jaeggi, John Jonides, Scott J. Peltier, Thad A. Polk, Patricia A. Reuter-Lorenz

**Affiliations:** ^1^Department of Psychology, University of Michigan, Ann Arbor, MI, United States; ^2^Department of Mental Health, Bloomberg School of Public Health, Johns Hopkins University, Baltimore, MD, United States; ^3^Department of Human Development and Family Science, Virginia Tech, Blacksburg, VA, United States; ^4^MIND Research Institute, Irvine, CA, United States; ^5^School of Education, University of California, Irvine, Irvine, CA, United States; ^6^Functional MRI Laboratory, Department of Biomedical Engineering, University of Michigan, Ann Arbor, MI, United States

**Keywords:** intrinsic activity, functional connectivity, graph theory, reliability analysis, intraclass correlation

## Abstract

Growing evidence suggests that healthy aging affects the configuration of large-scale functional brain networks. This includes reducing network modularity and local efficiency. However, the stability of these effects over time and their potential role in learning remain poorly understood. The goal of the present study was to further clarify previously reported age effects on “resting-state” networks, to test their reliability over time, and to assess their relation to subsequent learning during training. Resting-state fMRI data from 23 young (YA) and 20 older adults (OA) were acquired in 2 sessions 2 weeks apart. Graph-theoretic analyses identified both consistencies in network structure and differences in module composition between YA and OA, suggesting topological changes and less stability of functional network configuration with aging. Brain-wide, OA showed lower modularity and local efficiency compared to YA, consistent with the idea of age-related functional dedifferentiation, and these effects were replicable over time. At the level of individual networks, OA consistently showed greater participation and lower local efficiency and within-network connectivity in the cingulo-opercular network, as well as lower intra-network connectivity in the default-mode network and greater participation of the somato-sensorimotor network, suggesting age-related differential effects at the level of specialized brain modules. Finally, brain-wide network properties showed associations, albeit limited, with learning rates, as assessed with 10 days of computerized working memory training administered after the resting-state sessions, suggesting that baseline network configuration may influence subsequent learning outcomes. Identification of neural mechanisms associated with learning-induced plasticity is important for further clarifying whether and how such changes predict the magnitude and maintenance of training gains, as well as the extent and limits of cognitive transfer in both younger and older adults.

## Introduction

Aging is associated with cognitive decline that may be linked in part to altered communication among various brain regions (Reuter-Lorenz and Park, 2014). Indeed, aging has been shown to affect the integration of information both within and between functional brain networks (Ferreira and Busatto, 2013; Dennis and Thompson, 2014; Damoiseaux, 2017), which may have implications for cognitive performance. Despite accumulating evidence suggesting age effects on the configuration of large-scale functional brain networks (Achard and Bullmore, 2007; Meunier et al., 2009a; Onoda and Yamaguchi, 2013; Betzel et al., 2014; Cao M. et al., 2014; Chan et al., 2014; Song et al., 2014; Geerligs et al., 2015; Ng et al., 2016), the stability of these effects over time remains poorly understood. One goal of the present study was to clarify this issue by assessing age differences in functional network properties at two different time points.

A substantial body of evidence suggests that aging influences the functional organization of the brain, both globally and at the level of individual brain networks (reviewed in Ferreira and Busatto, 2013; Dennis and Thompson, 2014; Sala-Llonch et al., 2015; Damoiseaux, 2017). The functional organization of the brain has traditionally been studied using fMRI-based “resting-state” functional connectivity (Greicius et al., 2003; Power et al., 2011) and more recently, with graph-theoretic analyses (Bullmore and Sporns, 2009; Rubinov and Sporns, 2010). The graph-theoretic approach enables characterization of the brain's connectivity structure and derives measures that assess global and local features that may be important for network function (Bullmore and Sporns, 2009; Rubinov and Sporns, 2010). One such measure is modularity (Newman and Girvan, 2004; Newman, 2006), which indexes the extent to which a graph is organized into separate modules with dense within- and sparse between-modules connections, a fundamental principle thought to support the brain's functional segregation and integration (Dehaene et al., 1998; Sporns and Betzel, 2015). A number of prior investigations have identified lower modularity in aging (Onoda and Yamaguchi, 2013; Betzel et al., 2014; Cao M. et al., 2014; Song et al., 2014; Geerligs et al., 2015; but see Meunier et al., 2009a), with networks becoming less distinct due to increased between- and decreased within-module integration. This evidence is consistent with the idea of functional dedifferentiation (Park et al., 2004, 2010; Grady, 2012). Another set of measures characterizes the efficiency of information flow across the graph. Global efficiency indexes graph-wide integration and has been linked with the capacity for rapid information exchange among distributed regions, whereas local efficiency indexes integration at a regional level and has been linked with fault tolerance within specialized regions (Latora and Marchiori, 2003; Achard and Bullmore, 2007). Previous investigations have associated aging with lower local efficiency (Achard and Bullmore, 2007; Cao M. et al., 2014; Song et al., 2014; Geerligs et al., 2015), while global efficiency was reported to be similar irrespective of age (Cao M. et al., 2014; Song et al., 2014; Geerligs et al., 2015; but see Achard and Bullmore, 2007).

Importantly, differences in connectivity structure observed at a brain-wide level may be related to specific patterns at the level of individual networks, and current evidence suggests differential effects of aging on particular brain networks (Ferreira and Busatto, 2013; Dennis and Thompson, 2014; Sala-Llonch et al., 2015; Damoiseaux, 2017). Although the majority of investigations have targeted the default-mode network (DMN), showing lower functional connectivity between its different sub-components with aging (Andrews-Hanna et al., 2007; Damoiseaux et al., 2008), recent evidence also points to age effects in other brain networks, such as the cingulo-opercular/salience and sensorimotor networks (Meier et al., 2012; Onoda et al., 2012; He et al., 2014; Geerligs et al., 2015; La Corte et al., 2016)^1^. Thus, to complement information provided by brain-wide network assessments, metrics applied at the level of individual networks can also be employed. This includes the participation coefficient, which indexes the relation between intra- and inter-network connectivity for each node (Guimerà and Amaral, 2005).

In sum, although there are some inconsistencies across studies, available evidence points to lower within- and higher between-network connectivity with aging. This is expressed topologically as lower modularity, and is associated with lower local efficiency and preserved global efficiency, compared to younger age (see Damoiseaux, 2017 for a recent discussion). The first main goal of the present study was to assess the replicability of these previously reported age effects on functional network configuration.

Inconsistencies across investigations of age differences in network properties may stem from methodological differences but also from variability of network measures over time (van Wijk et al., 2010; Zalesky et al., 2016; Ciric et al., 2017; Geerligs et al., 2017). One way to assess reliability is by measuring the same subjects at two or more time-points, while using the same methodology, and quantifying the level of agreement between measurements by calculating the intraclass correlation coefficient (ICC) (Shrout and Fleiss, 1979; McGraw and Wong, 1996). A meta-analysis of test-retest reliability of graph-theoretic brain-network metrics identified overall good reliability (Welton et al., 2015). However, the available evidence related to aging is very limited. Investigations of age differences in network properties have typically used singular assessments, and hence the reliability of such effects over time is not clear (but see Geerligs et al., 2017). Thus, the second main goal of the present investigation was to extend the assessment of age differences in network properties to multiple time points within the same individuals and to evaluate reliability.

Clarification of age differences in network properties and their stability over time is important for further assessment of changes associated with cognitive training in older adults. Specifically, if aging influences relations between functional network properties and training outcomes, then these effects need to be disentangled from variability of network measures in the absence of intervention. Recent evidence suggests potential links between baseline properties of functional brain organization and benefits accrued over the course of cognitive training in older adults (Gallen et al., 2016a), although at this point such evidence is only preliminary. Although a growing body of studies suggests that some working memory (WM) interventions may alter functional network organization and have beneficial, albeit limited, effects on cognitive functioning (Buschkuehl et al., 2008; Lustig et al., 2009; Brehmer et al., 2014; Karbach and Verhaeghen, 2014; Stepankova et al., 2014; Ballesteros et al., 2015; Bherer, 2015; Mewborn et al., 2017; Román et al., 2017), evidence linking baseline functional network characteristics with training is limited (Arnemann et al., 2015; Gallen et al., 2016a). In one investigation of this topic, Gallen et al. (2016a) showed that older adults displaying greater network modularity at baseline also showed greater improvements in gist reasoning, following a strategic memory and reasoning training intervention (Vas et al., 2011). However, the potential role of other network properties in learning remains largely unknown. Thus, the third main goal of this investigation was to assess relations between baseline network properties and subsequent learning during training in older adults.

These questions were investigated in a sample comprising both healthy younger and older adults, using resting-state fMRI data acquired in 2 different sessions, both preceding a WM training intervention. A complete treatment of training outcomes and other behavioral data will be reported separately. Based on the extant evidence, we expected to find lower modularity and local efficiency in older compared to younger adults, and similar global efficiency across groups. We also expected these differences to be stable over time. Finally, the limited evidence linking network properties with training effects suggests that modularity is beneficial (Gallen et al., 2016a); therefore, we expected that network properties, in particular modularity (i.e., as reflected in the modularity index), would be linked to learning rates.

## Methods

### Participants

A sample of 23 younger (YA) and 23 healthy, cognitively normal older adults (OA) were recruited from the University of Michigan campus and community surrounding Ann Arbor, Michigan to participate in an adaptive verbal WM training study. All participants were right-handed, native English speakers with normal or corrected-to-normal hearing and vision and were screened for history of head injury, psychiatric illness, or alcohol/drug abuse. Data from 3 OA were excluded due to technical issues related to brain-imaging data acquisition. Thus, the sample for fMRI analyses consisted of 23 YA (age range: 18–28; 9 females) with a mean age (±S.D.) of 21.3 (±2.5) years and 20 OA (age range: 64–76; 9 females) with a mean age of 68.3 (±3.6) years. For analyses linking fMRI with behavioral results, 2 additional participants (1 OA) were excluded, due to technical issues related to behavioral task assessments, and thus these analyses were reported on 22 YA and 19 OA. Older adult participants completed the Short Blessed Test (Katzman et al., 1983) over the phone prior to inclusion in the study to screen for potential mild cognitive impairment, and additional neuropsychological assessments using the Montreal Cognitive Assessment (Nasreddine et al., 2005) confirmed normal cognitive function for all participants (scores ≥ 26). Additionally, participants were screened for depressive symptoms that could affect cognitive functioning using the depression module of the Patient Health Questionnaire (Kroenke et al., 2001). The University of Michigan Institutional Review Board approved all procedures, and all participants provided informed consent prior to participating.

### Imaging protocol

Functional MRI data were acquired during 8 min of resting state, following completion of a verbal WM task, in 2 sessions 2 weeks apart (t_1_, t_2_) (see Supplementary Figure 1 for an illustration of the study timeline). Participants were instructed to view a fixation cross in the center of the screen while keeping their mind calm and relaxed. Imaging data were collected using a 3 T General Electric MR750 scanner with an eight-channel head coil. Functional images were acquired in ascending order using a spiral-in sequence, with MR parameters: TR = 2,000 ms; TE = 30 ms; flip angle = 90°; field of view = 220 × 220 mm^2^; matrix size = 64 × 64; slice thickness = 3 mm, no gap; 43 slices; voxel size = 3.44 × 3.44 × 3 mm^3^. After an initial 10 s of signal stabilization, 235 volumes were acquired. A high-resolution T_1_-weighted anatomical image was also collected following the WM task and preceding resting-state acquisition, using spoiled-gradient-recalled acquisition (SPGR) in steady-state imaging (TR = 12.24 ms, TE = 5.18 ms; flip angle = 15°, field of view = 256 × 256 mm^2^, matrix size = 256 × 256; slice thickness = 1 mm; 156 slices; voxel size = 1 × 1 × 1 mm^3^). Images were de-spiked in *k*-space and reconstructed using an in-house iterative reconstruction algorithm with field-map correction (Sutton et al., 2003), which has superior reconstruction quality compared to non-iterative conjugate phase reconstruction.

### Preprocessing

Preprocessing was performed using SPM12 (Wellcome Department of Cognitive Neurology, London). Functional images were slice-time corrected, realigned, and co-registered to the anatomical image using a mean functional image. A study-specific anatomical template was created (younger and older adults together; Geerligs et al., 2015), using Diffeomorphic Anatomical Registration Through Exponentiated Lie Algebra (DARTEL) (Ashburner, 2007), based on segmented gray matter and white matter tissue classes, to optimize inter-participant alignment (Klein et al., 2009). The DARTEL flowfields and MNI transformation were then applied to the functional images and to the segments, and the functional images were resampled to 3 × 3 × 3 mm^3^ voxel size. To minimize artificial local spatial correlations, no additional spatial smoothing was applied (Salvador et al., 2005; Achard et al., 2006; Achard and Bullmore, 2007; Wang et al., 2010, 2011; Liao et al., 2011; Zalesky et al., 2012; Alakorkko et al., 2017).

Identification of outlier scans was performed using Artifact Detection Tools (ART; www.nitrc.org/projects/artifact_detect/), as follows. Scans were classified as outliers if frame-to-frame difference exceeded 0.5 mm in composite motion (combination of translational and rotational displacements) or 3 standard deviations in the global mean signal. On average, the proportion of outliers was below 5% in both YA (t_1_: 4.42%; t_2_: 2.72%) and OA (t_1_: 3.68%; t_2_: 3.74%). There were no significant differences between the two groups in the number of outlier scans (*p*'s > 0.4), or in the average (*p*'s > 0.1) or maximum (*p*'s > 0.5) motion, either before or after correcting for outlier scans (see “scrubbing” below).

### Graph construction

#### Functional connectivity analysis

Brain-wide functional connectivity analyses were performed using the Connectivity Toolbox (CONN; Whitfield-Gabrieli and Nieto-Castanon, 2012). To construct a brain-wide graph, we employed a commonly used functional atlas (Power et al., 2011), which comprises 264 meta-analytically defined coordinates, including cortical and subcortical areas; a 5 mm-radius sphere was centered at each of these coordinates. To ensure that the graph comprised regions that were not susceptible to fMRI signal drop-out, each sphere was filtered through a sample-level signal intensity mask, calculated as follows: First, binary masks were calculated for each subject, at each time point, thresholded at >70% mean signal intensity (Geerligs et al., 2015), computed over all voxels, using ART. Then, a sample-level mask was calculated, across all subjects and time points, using logical conjunction (see Supplementary Figure 2 for an illustration of the mask). Regions with fewer than 8 voxels (~50% volume) overlap with the sample-level mask were excluded, leaving 234 regions of interest (ROIs).

To remove physiological and other sources of noise from the fMRI time series we used linear regression and the anatomical CompCor method (Behzadi et al., 2007; Chai et al., 2012; Muschelli et al., 2014), as implemented in CONN. Each participant's white matter and cerebrospinal fluid segments, eroded by 1 voxel to minimize partial volume effects, were used as noise ROIs. The following temporal covariates were added to the model: signal extracted from each participant's noise ROIs (5 principal component analysis parameters for each^2^), motion parameters (3 rotation and 3 translation parameters, plus their first-order temporal derivatives), regressors for each outlier scan (i.e., “scrubbing”; one covariate was added for each outlier scan, consisting of 0's everywhere but the outlier scan, coded as “1”), and a session-onset regressor (a delta function convolved with the hemodynamic response function plus its first-order temporal derivative). The residual fMRI time series were band-pass filtered (0.01 Hz < *f* < 0.1 Hz). Pearson correlation coefficients were computed between the time courses of all pairs of functional ROIs, followed by Fisher-z transformation, and the diagonal of the connectivity matrix was set to zero. Graph construction and analyses were performed separately for each group and time point, using tools from the Brain Connectivity Toolbox (Rubinov and Sporns, 2010).

#### Group-level consensus partitions

**Figure 1:**
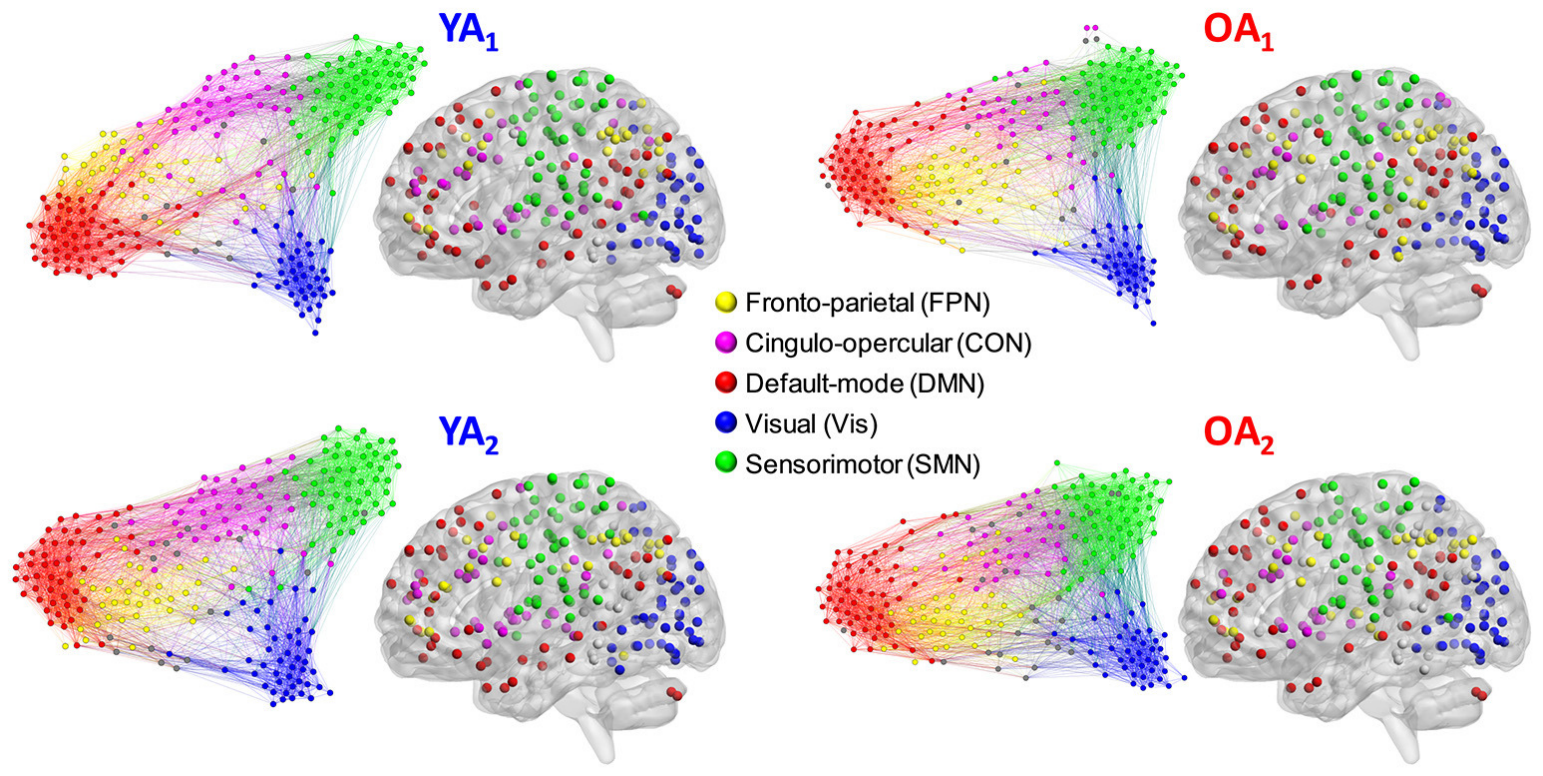
Representative group-level partitions. Functional networks were identified separately for each group and time point, using consensus partitioning. Five main modules were identified in both YA and OA, consistent with the main functional networks described in the literature (see main text for details). Nodes are color-coded by module, and within-module connections are displayed in the same color as the nodes. Nodes not belonging to the five main modules are displayed in gray. For illustration purposes, the force-directed graph displays 20% of the strongest connections and the anatomical projection displays nodes that form 2% of the strongest connections. The force-direct graph and anatomical projection were displayed using Gephi (http://gephi.org) and BrainNet Viewer (http://www.nitrc.org/projects/bnv/), respectively.

To achieve a community structure representative of each group, we used the Louvain community detection algorithm (Blondel et al., 2008), in conjunction with consensus clustering (Lancichinetti and Fortunato, 2012). This approach capitalizes on the consistency of each node's module affiliation across a set of partitions, to circumvent the known degeneracy of the Louvain algorithm (i.e., multiple partitioning solutions) (Good et al., 2010). To obtain a unique (i.e., threshold-independent) solution for each group, the Louvain algorithm was applied on weighted graph edges (positive only); see Cohen and D'Esposito (2016) for a similar approach. The group-level consensus partitions were employed to derive node–module assignments used for analyses at the level of individual modules/networks (see Network Measures sub-section below) and for display purposes (Figure 1).

Consensus clustering was applied first at the individual level, to generate a robust partition for each participant, and then at the group level, to generate a representative partition for each group and at each time point; see Dwyer et al. (2014) for a similar approach. First, to generate a robust partition for each participant, the Louvain algorithm was run 500 times. Because the algorithm is susceptible to local maxima, each initial partition was optimized using iterative community fine-tuning (Sun et al., 2009), which maximizes modularity by reassigning the nodes to modules and iterating the Louvain algorithm. For each participant, we constructed an agreement matrix representing the fraction of runs in which each pair of nodes was assigned to the same module. The Louvain algorithm was then iteratively run on the agreement matrix (500 Louvain runs at each step), to generate a consensus partition for each participant. For each iteration, the agreement matrix was recalculated and thresholded, until a single representative partition was obtained for each participant. Second, to generate a group-level representative partition, an agreement matrix was calculated based on the consensus partitions of all participants in one group. The Louvain algorithm was then run on the agreement matrix to obtain a consensus partition for each group, as described above. The resolution parameter of the Louvain community detection algorithm (γ) and the thresholding parameter for the agreement matrix (τ) were determined using a procedure that maximized modularity over all group-level partitions. Specifically, we ran the procedure described above for typical ranges of values for both parameters and chose those values that, on average, maximized modularity across all 4 group-level partitions (see below for a formal description of modularity). The value ranges were γ between 1 and 1.5 and τ between 0.2 and 0.5, with increments of 0.05 for each parameter. The maximum average modularity was *Q* = 0.71, achieved for γ = 1.25 and τ = 0.5, and these parameters were used for subsequent analyses.

#### Connection density thresholding

We used density-based thresholding, which equates the number of edges across graphs and allows proper between-groups comparisons (van Wijk et al., 2010; Garrison et al., 2015). To ensure that results were not due to any specific threshold, calculations were performed for a range comprising 2–10% of the strongest connections, in 1% increments. This threshold range is similar to that used in generating the Power et al. (2011) functional atlas and matches the range previously employed by Geerligs et al. (2015), thus enabling comparison of results. In general, stringent threshold ranges are preferable because inclusion of false-positive connections is more detrimental to network measures computation than exclusion of false-negative connections (Zalesky et al., 2016). The average number of disconnected nodes at each threshold in the 2–10% range was as follows: 47, 27, 17, 10, 7, 4, 3, 2, and 1. Because average connectivity was similar across groups (as assessed by permutation testing on positive edges; t_1, 2_: *p*'s > 0.2), density-based thresholding was likely unbiased across groups (Zalesky et al., 2016; van den Heuvel et al., 2017). To calculate network measures, connectivity values were binarized for each threshold (i.e., 1 if above, 0 if below threshold). Between-groups comparisons of graph-theoretic measures used binarized graphs and reported graph metrics are values averaged across all thresholds, unless specified otherwise.

### Network measures

To assess the strength of module segregation, we calculated the modularity index (*Q*) (Newman and Girvan, 2004; Newman, 2006), which compares the observed intra-module connectivity with that which is expected by chance. Higher modularity values indicate stronger separation of the graph's modules. The modularity index is formally defined as follows:

$$\begin{array}{l} Q=\frac{1}{2E}\sum_{ij}\left[A_{ij}-\gamma e_{ij}]\delta (m_{i},m_{j}\right) \end{array}$$

where *E* is the number of graph edges, *A* is the adjacency matrix, γ is the resolution parameter, *e* is the null model [*e* = *k*_*i*_*k*_*j*_/2*E*, where *k*_*i*_ and *k*_*j*_ are the degrees (i.e., number of connections) of the nodes *i* and *j*], and δ is an indicator that equals 1 if nodes *i* and *j* belong to the same module and 0 otherwise. The modularity score for each participant was calculated as the average over 500 runs of the Louvain algorithm with iterative community fine-tuning. For consistency with the consensus clustering procedure described above, the same resolution parameter (γ = 1.25) was used.

To assess the integration of information, we calculated global and local efficiency (Latora and Marchiori, 2003). Global efficiency indexes integration at the level of the entire graph and it is defined as follows:

$$\begin{array}{l} E_{glob}=\frac{1}{N(N-1)}\sum_{i\neq j}\frac{1}{L_{ij}} \end{array}$$

where *N* is the number of nodes in the graph and *L*_*ij*_ is the shortest path length between nodes *i* and *j*. By contrast, local efficiency is a node-specific measure, and is defined relative to the sub-graph comprising the immediate neighbors of a node. Local efficiencies for all nodes were averaged to provide an estimate of the mean local efficiency of the entire graph or of a module. Local efficiency of a node *i* is defined as follows:

$$\begin{array}{l} E_{loc}(i)=\frac{1}{N_{G_{i}}(N_{G_{i}}-1)}\sum_{j,h\in G_{i}}\frac{1}{L_{jh}} \end{array}$$

where *G*_*i*_ is the sub-graph comprising all the immediate neighbors of the node *i*.

Another node-specific measure is the participation coefficient (Guimerà and Amaral, 2005), which indexes inter-network connectivity by quantifying the distribution of each node's connections across different modules. Participation coefficients of all nodes within a module were averaged to provide an estimate of mean participation for a module. Participation coefficient of a node *i* is defined as follows:

$$\begin{array}{l} P\left(i\right)=1-\sum_{m=1}^{M}{\left[\frac{k_{i}(m)}{k_{i}}\right]}^{2} \end{array}$$

where *M* is the number of modules in the graph, and *k*_*i*_(*m*) is the degree of node *i* within its own module *m*, and *k*_*i*_ is the degree of node *i* regardless of module membership.

Finally, to assess the convergence of results based on the graph-theoretic measures described above with simpler connectivity analyses, we calculated within- and between-module connectivity using an approach similar to Geerligs et al. (2015). For completeness, this procedure was performed separately for positive and negative connectivity values. First, the initial connectivity matrices were thresholded by retaining values that survived a false discovery rate (FDR) correction (*q* < 0.05) (Benjamini and Hochberg, 1995) and setting all the other values to zero. Then, for each module and pair of modules, we computed the sum of all connectivity values and divided by the number of possible connections to estimate within- and between-modules connectivity. Of note, this procedure was used only for the analysis of within- and between-networks connectivity, and it did not influence the previously introduced graph-theoretic measures, which were all calculated on unweighted (i.e., binary) graphs.

### Statistical methods

As a general strategy, assessments were performed on metrics averaged across all thresholds, and significant results were followed-up with tests for each threshold, to assess consistency across the threshold range.

#### Age differences in community structure

To assess age differences in community structure, we compared module composition between groups using normalized mutual information (NMI) (Kuncheva and Hadjitodorov, 2004) and permutation testing. NMI measures how much information about the structure of one partition reduces uncertainty about the structure of another partition, and is a relative measure that varies from 0 (completely independent) to 1 (identical partitions). Because individual similarity measures are not independent, we used an unbiased procedure that compared the average between-groups similarity in the actual data with a null distribution based on randomizing group memberships; see Alexander-Bloch et al. (2012) for a similar approach. Between-groups similarity in the actual data was calculated for each density threshold, by averaging the pair-wise partition similarity for all subjects across the two groups, separately at each time point. For each subject, we used the partition with the highest modularity for each threshold, calculated over 500 Louvain repetitions with community fine-tuning and resolution γ = 1.25. The null distribution was calculated in a similar way, using the randomly divided groups over 5,000 permutations, while retaining original group sizes. If the actual between-groups NMI was smaller than the 5th percentile of the null distribution, the difference was considered significant. Furthermore, to determine whether one group showed more similar partitions than the other, we examined within-group partition similarity. This analysis was performed in a similar way, by averaging pair-wise partition similarity separately for subjects in each group. Finally, to examine differences in the stability of partitions over time, we calculated within-subject partition similarity over the two sessions. A between-group difference in partition similarity over time was tested directly, using permutation testing (Groppe et al., 2011).

#### Age differences in network measures

Age differences in network measures were first assessed brain-wide (modularity, global efficiency, and local efficiency) and then significant results were followed-up by analyses at the level of each module or network (participation coefficient and local efficiency). To ensure comparability at the level of individual networks, each module was represented only by those nodes that were consistently assigned to the same module, both across groups and time points, based on the group-level consensus partitions; see Geerligs et al. (2015) for a similar approach. Between-groups differences in network properties were assessed using permutation testing, and a family-wise error (FWE) correction for multiple comparisons based on the “max statistic” method (Blair and Karniski, 1993; Groppe et al., 2011) was applied to account for simultaneous testing of the five main modules identified (see Results section). As mentioned above, an ancillary analysis of within- and between-modules connectivity was also performed and the same FWE correction for multiple comparisons was applied for this analysis as well.

#### Reliability analysis

The intraclass correlation coefficient (ICC) was employed to measure the absolute agreement for each graph metric between the two sessions (McGraw and Wong, 1996; Welton et al., 2015). We used a mixed model^3^ ICC_(A, k)_ to estimate the degree of absolute agreement of measurements that are averages of *k* = 2 independent measurements on randomly selected subjects. ICC was calculated as follows: *ICC* = (*MS*_*R*_–*MS*_*E*_)/[*MS*_*R*_ + (*MS*_*C*_ – *MS*_*E*_)/*n*], where *MS*_*R*_ is mean square for rows/subjects, *MS*_*E*_ is mean square error, and *MS*_*C*_ is mean square for columns/assessments (Shrout and Fleiss, 1979; McGraw and Wong, 1996). We used the following guidelines for ICC interpretation: <0.20, poor; 0.21–0.40, fair; 0.41–0.60 moderate; 0.61–0.80 strong; >0.8, almost perfect (Montgomery et al., 2002; Telesford et al., 2010).

#### Links with learning during WM training

The second scanning session was followed by 10 days of computerized verbal WM training (Supplementary Figure 1). The adaptive training task consisted of a modified WM item-recognition task that required participants to encode and retain consonant letters of variable set size for several seconds (Sternberg, 1966; see also Stepankova et al., 2014); set size changed adaptively depending on participants' performance. Participants completed 6 blocks of 14 trials during each training session. Here, we focus on training-related improvements in WM performance specifically, as measured by mean set size achieved during each training session for each participant, to evaluate their relationship with network properties. Furthermore, we focused on early and late learning rates, defined as the performance change between training sessions 1 and 2 (*early* learning rate), and as the performance change across training sessions 2–10 (*late* learning rate), respectively, modeled for each individual using a linear spline term with a knot at the second training session (see Appendix for details). YA had a higher mean early slope than OA [*t*_(39)_ = 3.59, *p* = 0.001], but late slope did not differ by age group [*t*_(39)_ = 1.64, *p* = 0.109].

To assess links between network measures and learning rates, we calculated correlations between brain-wide network measures and early learning slopes, separately for each group and at each time point. We focused on early learning rates because age differences were identified in early but not in late learning slopes. Due to relatively small sample sizes, we employed Spearman's rank correlation coefficient (ρ) to minimize influence from extreme values. Significant brain-wide results were followed by assessments at the level of each module/network, corrected for multiple comparisons using the permutation-based “max statistic” method (Groppe et al., 2011). We took multiple steps to assess the robustness of our findings, using a procedure similar to Gallen et al. (2016a). First, to assess whether the relations between network measures and learning rates were constantly present over the threshold range, we tested these relations separately for each threshold. Second, given the absence of differences in motion across groups and time points (see Preprocessing sub-section above), we performed partial Spearman correlations (ρ_*p*_) to examine whether controlling for motion altered the relations between brain-wide network measures and learning rates.

## Results

### Age differences in community structure

**Figure 2:**
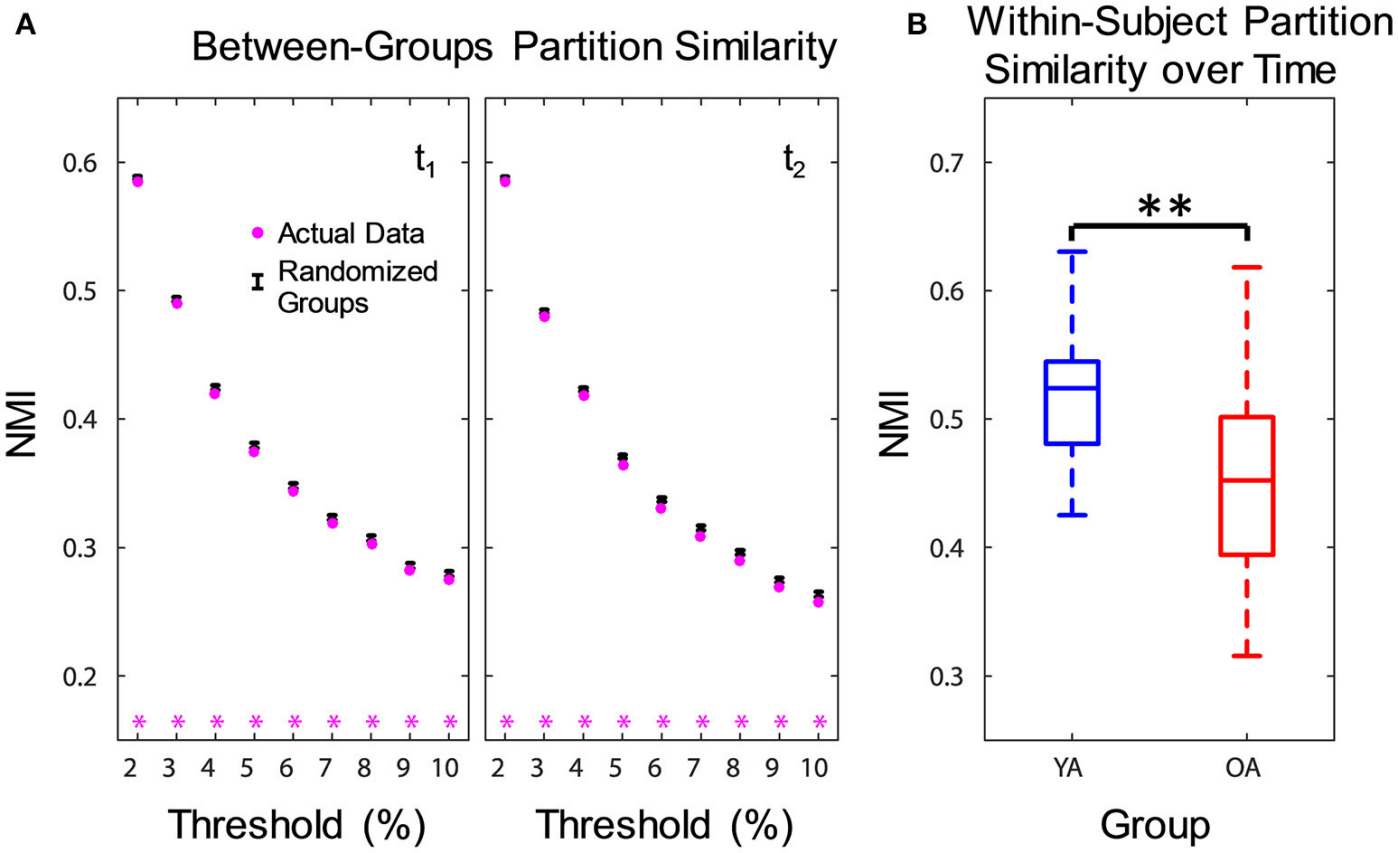
Age differences in community structure. Similarity of community structure between YA and OA was significantly lower than expected based on the permuted data, at both time points and consistently across all thresholds **(A)**. Also, OA showed less within-subject partition similarity across time **(B)**. Boxplots in the right panel depict values averaged across all thresholds. NMI, normalized mutual information; t_1_, time point 1; t_2_, time point 2; YA, younger adults (blue color); OA, older adults (red color). Magenta asterisks indicate *p* < 0.05 for each threshold. ^**^p < 0.01, across all thresholds.

Functional networks were identified separately for each group and time point, using consensus partitioning (see Methods section for details). Similar modules were identified in both YA and OA, consistent with the main functional networks described in the literature (Power et al., 2011; Yeo et al., 2011): fronto-parietal (FPN), cingulo-opercular/salience (CON), default-mode (DMN), visual (Vis) and somato-sensorimotor (SMN) (Figure 1). The community structure of the partitions for each age group was examined using normalized mutual information (NMI). Results showed differences in node-module assignment between YA and OA, at both time points (Figure 2). First, analysis of *between-groups* partition similarity showed that similarity of community structure between YA and OA was significantly lower than expected based on the permuted data (t_1, 2_: *p*'s < 0.001; Figure 2A). Second, analysis of *within-group* partition similarity showed less similarity for OA as a group. Specifically, partition similarity for YA was higher (t_1_: *p* = 0.003; t_2_: *p* < 0.001) whereas for OA was lower (t_1_: *p* = 0.007; t_2_: *p* = 0.003) than expected based on the permuted data (Supplementary Figure 3). This indicates that there is greater heterogeneity in OA's partitions, i.e., their partitions are less similar to one another than YA's partitions. Finally, analysis of *within-subject* similarity across time showed less within-subject consistency for OA (*p* = 0.001; Figure 2B), indicating more variability in node-module assignment in OA across time. In summary, although similar functional networks were identified in both YA and OA, their composition differed between groups, and OA showed less similarity, both as a group and across time, compared to YA.

### Age differences in network measures and reliability analysis

To complement the comparisons of community structure presented above, we assessed age differences in several network measures. Network measures were first calculated brain-wide, followed by an assessment of their reliability over time. Then, significant brain-wide differences were followed-up by assessments at the level of each of the five modules/networks.

#### Brain-wide network measures

**Figure 3:**
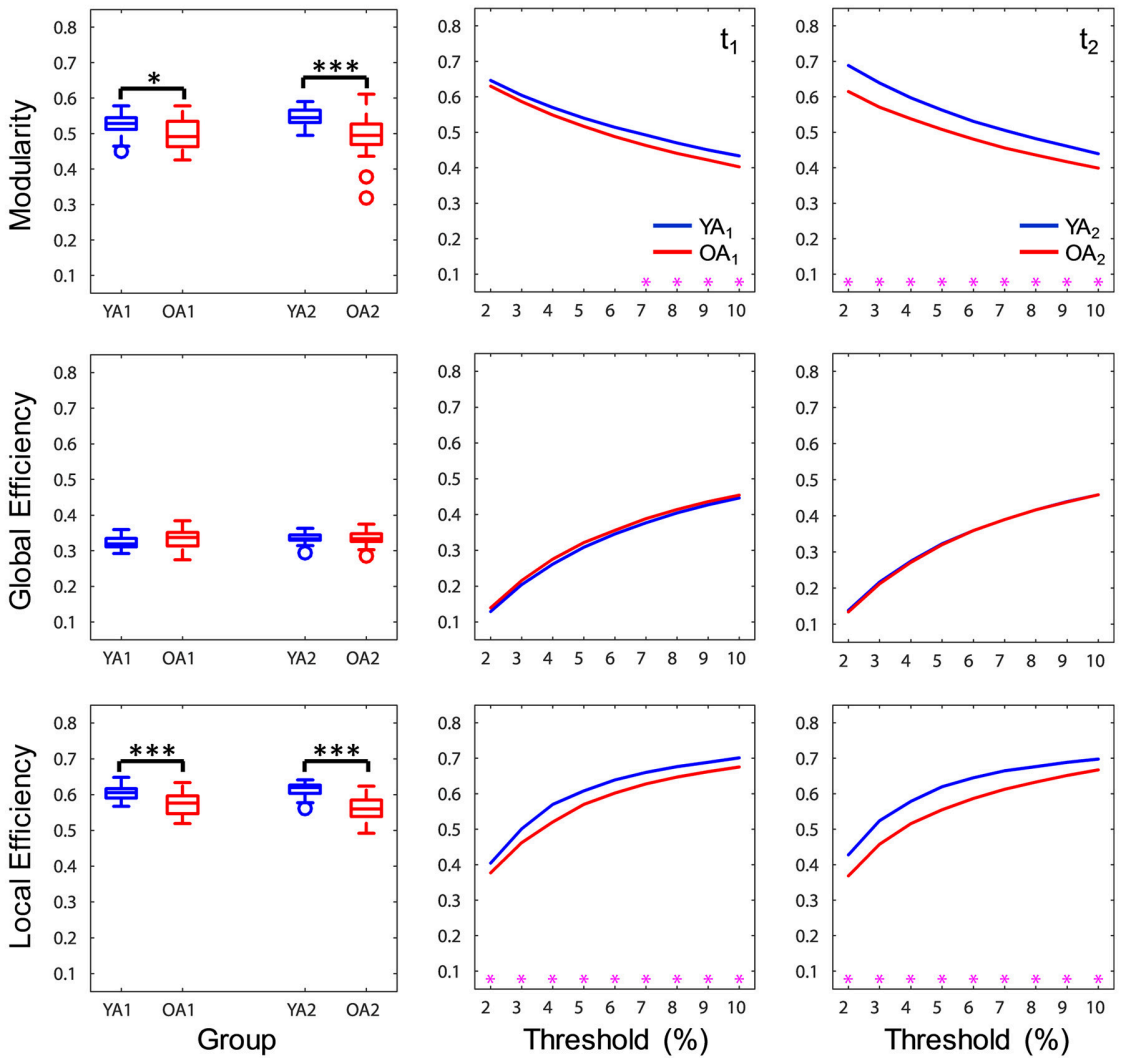
Age differences in brain-wide network measures. At a brain-wide level, OA showed lower modularity and local efficiency compared to YA, whereas global efficiency was not significantly different across groups. Boxplots in the left panel depict values averaged across all thresholds. YA, younger adults (blue color); OA, older adults (red color); t_1_, time point 1; t_2_, time point 2. Magenta asterisks indicate *p* < 0.05 for each threshold. ^*^p < 0.05, ^***^p < 0.001, across all thresholds.

At a brain-wide level, OA showed lower modularity indices at both time points (t_1_: *p* = 0.046, t_2_: *p* < 0.001), indicating lower intra-module connectivity compared to YA. Furthermore, OA consistently showed lower local efficiency (t_1, 2_: *p*'s < 0.001), while global efficiency was not significantly different across groups (t_1, 2_: *p*'s > 0.1), suggesting age differences in local but not global integration of information (Figure 3). Ancillary correlation analyses between age and brain-wide network measures within the OA group revealed no significant results (*p*'s > 0.05).

#### Reliability analysis of brain-wide network measures

**Figure 4:**
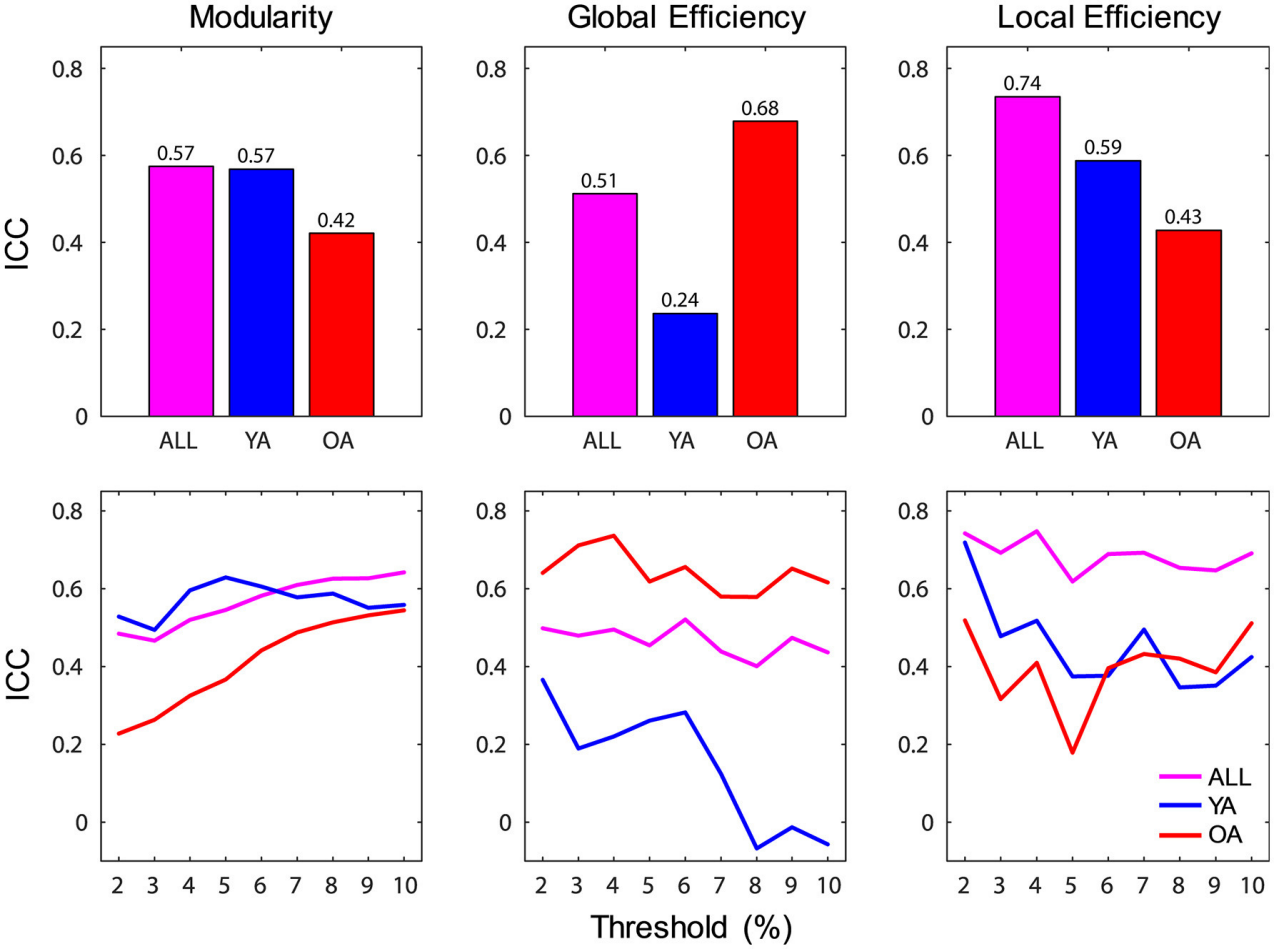
Reliability of brain-wide network measures. **(Top)** Brain-wide measures showed overall moderate to strong ICC over time, and for each group the agreement ranged from fair (>0.2) to strong (>0.6); bar graphs depict ICC values calculated across all thresholds. **(Bottom)** Reliability of network measures was generally stable across thresholds, with the exception of global efficiency for YA; line graphs depict ICC values calculated for each threshold. ICC, intraclass correlation coefficient; ALL, all subjects (magenta color); YA, younger adults (blue color); OA, older adults (red color).

Reliability of brain-wide network measures was assessed using intraclass correlation (ICC), by calculating the absolute agreement of each graph metric across sessions. Brain-wide measures showed overall moderate to strong ICC over time (range 0.51–0.74), with the highest agreement for local efficiency (Figure 4). For each group, the agreement ranged from fair (>0.2) to strong (>0.6), with YA showing lowest agreement for global efficiency. Examination of the profiles of ICC values across the range of thresholds indicated that the reproducibility of network measures was generally stable across thresholds, with the exception of global efficiency for YA.

#### Individual network measures

**Figure 5:**
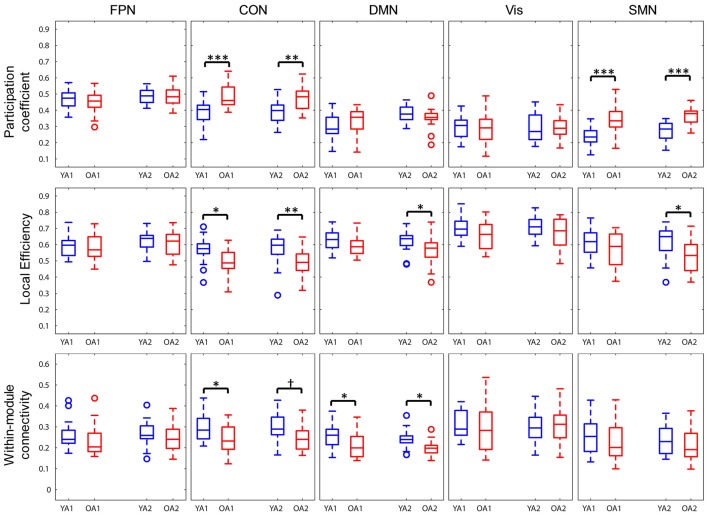
Age differences in individual network measures. OA showed greater participation coefficients for CON and SMN, lower local efficiency for CON, and for DMN and SMN only at t_2_, and lower within-module connectivity within DMN and within CON, compared to YA. FPN, fronto-parietal network; CON, cingulo-opercular network; DMN, default-mode network; Vis, visual network, SMN, somato-sensorimotor network; YA, younger adults (blue color); OA, older adults (red color). ^*^p < 0.05, ^**^p < 0.01, ^***^p < 0.001, across all thresholds and corrected for multiple comparisons. ^†^*p* = 0.016, across all thresholds, uncorrected.

Network properties were also assessed at the level of each individual network (Figure 5). To ensure comparability across groups and time points, each network was represented by only those nodes that were consistently assigned to the same network, both across groups and time points (see Methods section for details). OA showed greater participation coefficient for CON (t_1_: *p*_*FWE*_ < 0.001; t_2_: *p*_*FWE*_ = 0.002) and SMN (t_1, 2_: *p*_*FWE*_'s < 0.001), indicating that, compared to YA, a larger proportion of the nodes in these networks had connections outside the networks they belonged to. OA also showed lower local efficiency for CON (t_1_: *p*_*FWE*_ = 0.014; t_2_: *p*_*FWE*_ = 0.008) at both time points, and for DMN (*p*_*FWE*_ = 0.029) and SMN (*p*_*FWE*_ = 0.01) at t_2_. We also examined within- and between-network connectivity, using a procedure similar to Geerligs et al. (2015). Regarding *within*-networks connectivity, OA showed lower connectivity within DMN (t_1_: *p*_*FWE*_ = 0.04; t_2_: *p*_*FWE*_ = 0.018) and within CON (*p*_*FWE*_ = 0.035) at t_1_, compared to YA. Regarding *between*-networks connectivity, OA showed greater positive connectivity between FPN and SMN (*p*_*FWE*_ = 0.005) and between CON and SMN (*p*_*FWE*_ = 0.048), as well as lower negative connectivity (anticorrelation) between CON and SMN (*p*_*FWE*_ = 0.043), at t_2_. No other age differences in between-networks connectivity survived FWE correction for multiple comparisons. Ancillary correlation analyses between age and individual network measures within the OA group identified a significant negative correlation between age and within-DMN connectivity at t_1_ (ρ = −0.65, *p*_*FWE*_ = 0.015).

In summary, OA showed lower brain-wide modularity and local efficiency compared to YA, with the difference in local efficiency showing most consistency across time. At the level of individual networks, CON showed substantial differences between groups, reflected in all examined properties. Additionally, DMN and SMN were characterized by lower intra-network connectivity and greater participation, respectively, in OA.

### Links with learning during WM training

To assess links between network measures and performance during cognitive training, we calculated Spearman correlation coefficients, separately for each group and at each time point. Similar to the assessment of age differences in network measures, significant brain-wide results were followed by analyses of robustness and assessments at the level of individual networks.

#### Brain-wide network measures

**Figure 6:**
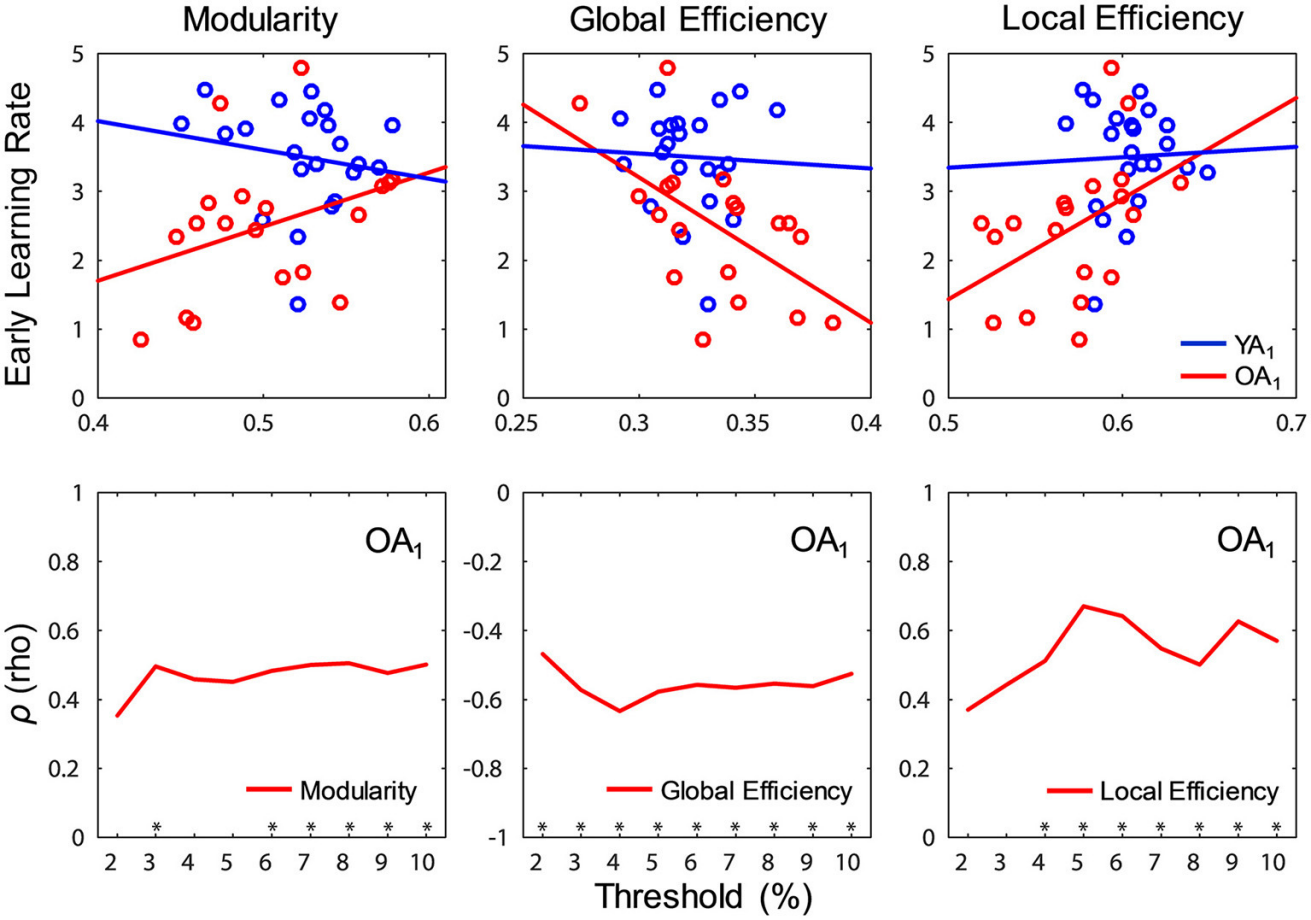
Relations between network measures and learning during WM training. **(Top)** Modularity (ρ = 0.51, *p* = 0.028) and local efficiency (ρ = 0.59, *p* = 0.01) were positively correlated, whereas global efficiency (ρ = −0.61, *p* = 0.007) was negatively correlated with early learning rates in OA (red color), only at t_1_; blue and red lines show least squares lines. **(Bottom)** Relations between network measures and learning rates (Spearman correlation) were fairly constant across thresholds. t_1_, time point 1; YA, younger adults (blue color); OA, older adults (red color). ^*^p < 0.05, for each threshold.

Interestingly, significant relations between network measures and learning rates were detected only for OA and only at t_1_. Specifically, modularity (ρ = 0.51, *p* = 0.028) and local efficiency (ρ = 0.59, *p* = 0.01) were positively correlated with early learning rates, whereas global efficiency (ρ = −0.61, *p* = 0.007) was negatively correlated with early learning rates (Figure 6, top panel). Ancillary analyses were performed to test for influences of educational level and sex on these results. There were no significant correlations between the number of years of education and networks measures (*p*'s > 0.5), and controlling for the number of years of education did not substantially influence the relations between network measures and learning rates. Also, Spearman correlations performed separately by sex showed similar trends in both males and females, and there were no sex differences in correlation strengths (*p*'s > 0.6).

#### Robustness analysis

We took multiple steps to assess the robustness of our findings, using a procedure similar to Gallen et al. (2016a). First, we assessed whether the relations between network measures and learning rates were constantly present over the threshold range, and the results confirmed that all these relations were fairly consistent across thresholds (Figure 6, bottom panel). Second, given the absence of differences in motion across groups and time points (see Methods section), controlling for motion (i.e., partial correlations) did not substantially alter the relations between any of the brain-wide network measures and learning rates (modularity: ρ_*p*_ = 0.49, *p* = 0.04; local efficiency: ρ_*p*_ = 0.55, *p* = 0.019; global efficiency: ρ_*p*_ = −0.59, *p* = 0.01).

#### Individual network measures

To further elucidate the relations between network characteristics and early learning rates, significant results at the brain-wide level were followed-up by analyses at the level of individual networks. The results showed that participation of CON at t_1_ was negatively correlated with learning rates in OA (ρ = −0.81, *p*_*FWE*_ < 0.001), consistent with the brain-wide results. No other correlations survived FWE correction for multiple comparisons.

In summary, brain-wide network measures at t_1_ were linked to learning rates during training in OA but not in YA. At the level of individual networks, participation of CON showed links with training effects consistent with the patterns identified by the brain-wide analyses.

## Discussion

The goals of the present investigation were to assess the replicability of previously reported age effects on resting-state networks, to examine their reliability over time, and to assess their relation to behavioral outcomes (namely learning rates during a cognitive training intervention). Similar to previous investigations, we identified both consistencies in network structure and differences in module composition between groups. Notably, OA showed less similarity of their network partitions compared to YA, both as a group and across time. Regarding brain-wide network measures, OA showed lower modularity and local efficiency compared to YA, with the difference in local efficiency showing most consistency across time. At the level of individual networks, OA showed substantial differences in CON, reflected in all examined metrics, as well as lower intra-network connectivity in DMN and greater participation of SMN. Finally, baseline brain-wide network measures were linked to early learning rates in OA but not in YA, and the participation of CON showed links with early learning rates consistent with the patterns identified by the brain-wide analyses. The main findings are discussed, in turn, below.

The present results replicate previously reported age differences in functional network properties (Achard and Bullmore, 2007; Meunier et al., 2009a; Onoda and Yamaguchi, 2013; Betzel et al., 2014; Cao M. et al., 2014; Song et al., 2014; Geerligs et al., 2015) and extend these findings to multiple time points (Welton et al., 2015). Regarding community structure, the present results showing age differences in module composition, but overall similar modules are consistent with previous evidence (Geerligs et al., 2015) and suggest age-related topological changes in the context of overall similar functional configuration, irrespective of age. Furthermore, the results showing less similarity of network partitions in OA, both as a group and across time, are in line with recent evidence suggesting reduced baseline stability of network activity with aging (Tsvetanov et al., 2016).

Regarding age differences in network measures, we identified reliable age differences in brain-wide modularity and local efficiency, consistent with previous investigations (Achard and Bullmore, 2007; Onoda and Yamaguchi, 2013; Betzel et al., 2014; Cao M. et al., 2014; Song et al., 2014; Geerligs et al., 2015). Modularity indexes the degree to which a graph can be partitioned into multiple communities, and is considered a central principle of brain organization, supporting functional segregation and integration through communication within- and between-modules, respectively (Dehaene et al., 1998; Sporns et al., 2000; Meunier et al., 2009b; Sporns and Betzel, 2015). Thus, results showing lower modularity in OA compared to YA suggest loss of functional specificity of the brain networks with aging (Ferreira and Busatto, 2013; Damoiseaux, 2017; Naik et al., 2017). Global efficiency indexes graph-wide integration and has been linked with information exchange among distributed regions, whereas local efficiency indexes regional-level integration and has been linked with fault tolerance within specialized areas (Latora and Marchiori, 2003; Achard and Bullmore, 2007). In general, the argument is that brains maximize cost-efficiency by favoring dense short-range connections and sparse long-range connections, because the latter are more costly (Achard and Bullmore, 2007; Bullmore and Sporns, 2012). Thus, results showing lower local efficiency in OA compared to YA suggest a reduction of cost-efficiency in aging; under conditions of similar connection density, which is considered a proxy for wiring cost, efficiency is lower in OA compared to YA (Achard and Bullmore, 2007; Geerligs et al., 2015). It should be noted, however, that wiring costs can only be approximated in functional networks, because two functionally connected regions do not necessarily share a direct structural link (Rubinov and Sporns, 2010; Zalesky et al., 2012). In fact, modularity and local efficiency are related measures, such that a system with denser local connections also tends to be more modular (Bullmore and Sporns, 2012). On the other hand, similar global efficiency irrespective of age has been explained by a greater number of inter-module connections in OA; specifically, more inter-module connections may counterbalance less intra-module connections, resulting in similar amounts of shortest path lengths between distant nodes (Song et al., 2014; Geerligs et al., 2015). In sum, these findings are consistent with overall patterns of decreased within- and increased between-system connectivity, suggesting decreased “system segregation” in aging (Betzel et al., 2014; Chan et al., 2014; Ferreira et al., 2016).

The present findings may also be relevant for better understanding task-related neural over-activation in OA relative to YA, which has been linked with both compensation and dedifferentiation (Cabeza, 2002; Park et al., 2004, 2010; Davis et al., 2008; Grady, 2008; Reuter-Lorenz and Cappell, 2008; Reuter-Lorenz and Park, 2014). Task-related over-activation in OA may be related to altered intrinsic network dynamics, reflected in differences in modularity and local efficiency “at rest.” Whereas the loss of functional specificity in aging (reflected by the decline in modularity) is consistent with the idea of dedifferentiation, reduced cost-efficiency (reflected by the decline in local efficiency) may be linked to compensatory processes that are overall less efficient than the primary computations (Reuter-Lorenz and Park, 2014). Thus, dedifferentiation and compensation may both be expressions of the same process of functional recalibration due to declining structure with aging (Naik et al., 2017). This also highlights the critical need for better integrating resting-state and task-related approaches, in order to develop a practical understanding of neurocognitive function and age-related change (Iordan and Reuter-Lorenz, 2016; see also Gallen et al., 2016b).

To assess the reliability of age differences in network properties, in the present study we measured the same participants over 2 sessions 2 weeks apart and calculated ICC of network properties between the 2 sessions (McGraw and Wong, 1996). Results showed consistent age differences in network properties over time, with overall strong to moderate ICCs, comparable to previous investigations (Telesford et al., 2010; Wang et al., 2011; Braun et al., 2012; Park et al., 2012; Cao H. et al., 2014; Welton et al., 2015), thus suggesting that the observed age differences are reliable. Interestingly, results showed relatively higher reliability for local compared to global efficiency (see also Park et al., 2012). This effect was driven by YA, who showed more global efficiency variability between the 2 sessions, and it might have been linked to residual effects from the WM tasks performed prior to the resting-state recordings (Barnes et al., 2009; Breckel et al., 2013; Gordon et al., 2014). In line with our findings, a study by Park et al. (2012) also identified low reliability of global efficiency in a test-retest investigation of resting-state data in YA, assessed over a 24-h period. The authors concluded that this was likely due to high variability of long-range connections (given the dependence of global efficiency on this topological feature), and may reflect greater influence of cognitive control on this measure, compared to local efficiency (Honey et al., 2009).

The results showing age differences in network properties at the level of individual modules complement and further elucidate the patterns of brain-wide results. Although DMN has traditionally been the most investigated resting-state network (Ferreira and Busatto, 2013; Damoiseaux, 2017), recent investigations also point to CON changes as prominent features of healthy aging (Meier et al., 2012; Onoda et al., 2012; He et al., 2014; La Corte et al., 2016). The cingulo-opercular network (or salience network, in alternative taxonomies) is anchored in the anterior cingulate and frontal operculum/anterior insula regions, and has been implicated both in stable set-maintenance (Dosenbach et al., 2006, 2007, 2008; Power and Petersen, 2013) and multimodal sensory integration (Seeley et al., 2007; Bressler and Menon, 2010; Menon, 2011). The present results, showing both higher participation coefficients and lower local efficiency and intra-module connectivity for CON in OA, suggest age-related dedifferentiation of this network and support the idea of changes in CON functionality as a hallmark of healthy aging (Meier et al., 2012; Onoda et al., 2012; He et al., 2014; La Corte et al., 2016). Greater participation coefficients for CON and SMN in OA indicate greater propensity of the nodes within these two networks to form links outside their own modules, and suggest that CON and SMN may drive the observed age differences in brain-wide modularity. Furthermore, local efficiency in CON was also consistently lower in OA, suggesting an age-related decline in local integration of information at the level of this network. In addition to CON, DMN showed consistent lower intra-module connectivity in OA relative to YA, in line with previous evidence (Andrews-Hanna et al., 2007; Damoiseaux et al., 2008; Ferreira and Busatto, 2013; Geerligs et al., 2015; Grady et al., 2016; Damoiseaux, 2017). Interestingly, our results did not show greater FPN-DMN inter-network connectivity in OA relative to YA (Geerligs et al., 2015; Turner and Spreng, 2015), which might have been related to the inclusion of relatively younger, high-functioning OA in our sample. Supporting this interpretation, a recent longitudinal study in OA (Ng et al., 2016) identified a u-shaped trajectory in which FPN-DMN inter-network connectivity initially *de*creased and then *in*creased with age, with a turning point around 65–70 years of age. An alternative interpretation is that the functional interactions between FPN and DMN in OA might have been influenced by residual task-effects, as outlined above.

Regarding links between network measures and learning rates during training, the present results showed that higher resting-state modularity and local efficiency, as well as lower global efficiency prior to training, were associated with better early learning in OA. Early learning rates are thought to reflect the initial attainment of peak performance within an individual's baseline performance range, rather than plasticity *per se* (Lövden et al., 2010). Notably, associations between network properties and early learning rates were observed only for OA and only at t_1_. While the presence of these associations only in OA could be interpreted in line with evidence pointing to age-related dissociations in the relations between network efficiency and cognitive performance (Stanley et al., 2015), the lack of consistency of these relations across time might be attributable to differences in residual task-effects related to the phenomenon of task exposure which may have, in turn, influenced the reliability of network measures across time. Specifically, if task exposure altered strategies for WM task performance across the two sessions for older but not for younger adults, the resting-state activity, which was always recorded subsequent to task performance, may have been differentially affected. Future analyses comparing the effects of task exposure on differences between task-related and subsequently recorded resting-state network configurations are needed to further clarify this aspect of the results.

Although evidence linking network properties with benefits accrued over the course of cognitive training is scarce, the present results are in line with previous findings showing positive relations for modularity in OA (Gallen et al., 2016a) and in patients with traumatic brain injury (Arnemann et al., 2015). Consistent with the idea that modularity supports both functional segregation and integration, previous evidence has positively linked modularity with cognitive performance (Stevens et al., 2012; Sadaghiani et al., 2015), and thus greater modularity during resting-state may reflect a more “optimal” functional organization that promotes cognitive improvements with training (Gallen et al., 2016a). Results at the level of individual networks add specificity to this interpretation, by associating lower CON participation coefficients with higher learning rates in OA. Combined with evidence showing greater participation coefficients for this network in OA as a group, the present findings provide preliminary evidence for a link between preserved CON segregation and better learning in OA.

The present results linking network properties at rest with learning rates can be interpreted in the light of evidence from investigations of task-related performance (Stanley et al., 2015; Cohen and D'Esposito, 2016; Bolt et al., 2017). Specifically, investigations comparing network properties across resting and task contexts have shown that cognitive task states are characterized by overall *lower* modularity and local efficiency, as well as *greater* global efficiency, and that such levels are positively associated with cognitive performance at the individual level in YA (Cohen and D'Esposito, 2016; Bolt et al., 2017). Consistent with this evidence, age-related investigations have linked lower local efficiency during task performance with better WM performance irrespective of age, whereas greater global efficiency was associated with better WM performance in YA but relatively worse WM performance in OA (Stanley et al., 2015). By contrast, prior investigations (Gallen et al., 2016a), as well as the present results, point to a seemingly inverse pattern characterizing the relationship between modularity “at rest” and learning rates in OA, whereby *greater* modularity and local efficiency, as well as *lower* global efficiency “at rest”, are associated with better learning. Although relations between resting and task-related network configurations are still not well understood, the present evidence suggests that the potential for dynamic network reconfiguration across different states might play an important role for understanding cognition and its plasticity in aging (Cole et al., 2014, 2016; Krienen et al., 2014; Iordan and Reuter-Lorenz, 2016). However, the exploratory nature of these findings advises their interpretation with caution.

### Limitations and future directions

Reliance on extreme groups to understand effects of aging has clear limitations, and thus future work assessing a broader age range (e.g., Chan et al., 2014), as well as longitudinal assessments of the same individuals over periods of years (e.g., Ng et al., 2016), are necessary to provide more comprehensive insights. Regarding the timing of resting-state acquisition, whereas a 6-min break from a preceding task can be a sufficient “wash-out” period for certain individuals under certain task conditions (Breckel et al., 2013), it is not as efficient as longer breaks, and thus resting-state recording before any task should be preferred. Finally, our investigations at the level of individual modules have been partly exploratory. Future studies with strong a priori hypotheses are needed to further elucidate effects of aging on specific within- and between-networks interactions.

## Conclusions

In conclusion, we successfully replicated previously reported age effects on resting-state networks, demonstrated their reliability over time, and identified links with initial learning during WM training. We identified both consistencies in network structure and differences in module composition between YA and OA, suggesting topological changes and less stability of functional network structure with aging. Lower modularity and local efficiency in OA suggests age effects on both functional segregation and integration of brain networks, consistent with the idea of age-related functional dedifferentiation. Importantly, these differences were replicable over time, with the difference in local efficiency showing most consistency. On the other hand, global efficiency did not differ between the two age groups and showed low reliability in YA. At the level of individual networks, specific differences were identified for CON, DMN, and SMN, suggesting age-related differential effects at the level of specialized brain modules. Finally, associations between network properties and early learning rates were identified for OA only at t_1_, suggesting that baseline network configuration may be informative in predicting aspects of learning in OA, albeit with some limitations. The present findings advance our understanding of the effects of aging on the brain's large-scale functional organization and provide preliminary evidence for network characteristics associated with learning during training. Continued identification of neural mechanisms associated with training-induced plasticity is important for further clarifying whether and how such changes predict the magnitude and maintenance of training gains, as well as the extent and limits of cognitive transfer in both younger and older adults.

## Author contributions

PR-L, JJ, TP, MB, SJ, BK, KC, KM, and SP designed the study. KC and KM collected the behavioral and brain imaging data, and analyzed the behavioral data. AI analyzed the resting-state brain imaging data and wrote the original draft. All authors reviewed and edited the final manuscript.

### Conflict of interest statement

MB is employed at the MIND Research Institute, whose interest is related to this work. SJ has an indirect financial interest in the MIND Research Institute. The other authors declare that the research was conducted in the absence of any commercial or financial relationships that could be construed as a potential conflict of interest.

## Footnotes

^1^While graph theory has been typically employed to assess global and local measures of connectivity, much evidence regarding aging effects on specific brain networks has been derived using complementary approaches, such as seed-based functional connectivity and independent component analysis. Although these approaches differ in important ways (see Ferreira and Busatto, 2013; Dennis and Thompson, 2014 for recent discussions), results have been overall convergent (see Geerligs et al., 2015 for a recent graph theory investigation at the level of individual networks).

^2^Regressing out multiple principal components from noise ROIs typically leads to better noise correction than regressing out the average noise signal because physiological noise (including motion) is not spatially homogeneous across the brain (Chai et al., 2012; Muschelli et al., 2014).

^3^We made no assumption of interchangeability of t_1_ and t_2_ assessments because the resting-state data were acquired following completion of a verbal WM task inside the scanner, and thus potential differences in task performance at the two time points might have differentially influenced resting-state recordings. We also expected, however, that these effects would be mitigated by a ~6 min break (recording the T_1_-weighted anatomical image) following the WM task and preceding the resting-state acquisition (Breckel et al., 2013).

## Appendix

### Learning rates calculation

Participants completed 6 blocks of 14 trials during each of the 10 training sessions (s_1_-s_10_). The number of letters in each memory set (i.e., set size) remained constant for each block and was determined by the participant's performance in the previous block. The set size increased by one letter if participants' accuracy was >93% on the preceding block and decreased by one letter if their accuracy was <70%. Thus, for each participant, we calculated the average set size across the 6 blocks for each session.

Learning rates were calculated using those average set-sizes achieved during each training session for each participant. According to Lövden et al. (2010), early learning is thought to reflect the initial attainment of peak performance within an individual's baseline performance range, and is therefore associated with a steep slope. Here, we defined *early* learning rate as the performance change (i.e., slope) between training sessions 1 and 2. On the other hand, later learning requires prolonged training, and is thought to reflect plasticity. Thus, we used the average performance change (i.e., slope) across training sessions 2–10 to reflect *later* learning rate.

Early and late learning rates were modeled for each individual using a linear spline term with a knot at training session 2 (s_2_). The knot location was selected by first fitting a negative exponential growth curve to the observed data using the NLME package in R (Pinheiro and Bates, 2000; Rhodes and Katz, 2017). This procedure was used as an alternative to the assignment of knot location based on only visual inspection of the overall training performance curves. The equation for the negative exponential growth curve is: *y*(*t*) = φ_3_– (φ_3_ − φ_1_) exp[-φ_2_ * *t*], where *t* represents the training session, φ_1_ is the performance at asymptote, φ_2_ is performance at session 1 (*t* = 0), and φ_3_ is the growth rate. The growth rate parameter can be used to determine the inflection point of the curve, which is the session by which half of the total growth has occurred, according to the formula: *t*_(0.5)_ = log 2/exp(φ_3_). The parameter estimates for the observed data were φ_1_ = 9.0 (*SE* = 0.11), φ_2_ = 5.0 (*SE* = 0.23), φ_3_ = -0.17 (*SE* = 0.14). Using the equation above, the inflection point occurred slightly before s_2_ (*t* = 0.8), and was similar across both age groups. Individual linear spline models with a knot at s_2_ were then computed for each participant.
