# Supplementary material for: Aging and Network Properties: Stability Over Time and Links with Learning during Working Memory Training
Source: Front Aging Neurosci. 2018 Jan 4;9:419. doi: 10.3389/fnagi.2017.00419 (PMC5758500; doi:10.3389/fnagi.2017.00419)
Supplement: Supplementary file 1 [file Image1.PDF]

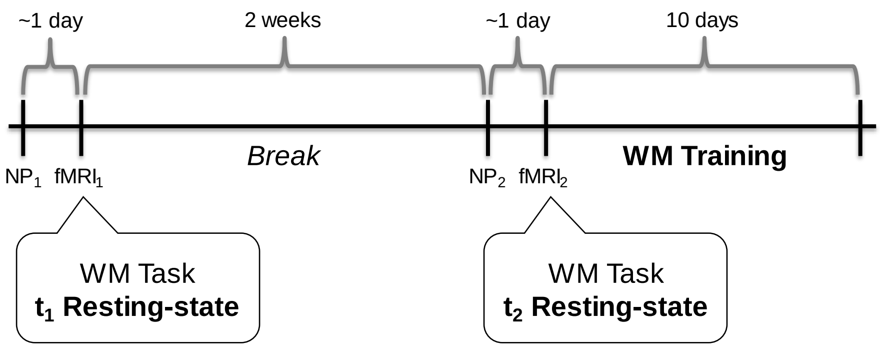

Supplementary Figure 1. Study Timeline. Resting-state fMRI data were acquired following completion of a verbal WM task, in 2 sessions 2 weeks apart (t_1, t_2), followed by 10 days of computerized WM training. Data reported here is depicted in bold. NP, neuropsychological testing; fMRI, functional MRI session.
