# Supplementary material for: Aging and Network Properties: Stability Over Time and Links with Learning during Working Memory Training
Source: Front Aging Neurosci. 2018 Jan 4;9:419. doi: 10.3389/fnagi.2017.00419 (PMC5758500; doi:10.3389/fnagi.2017.00419)
Supplement: Supplementary file 2 [file Image2.pdf]

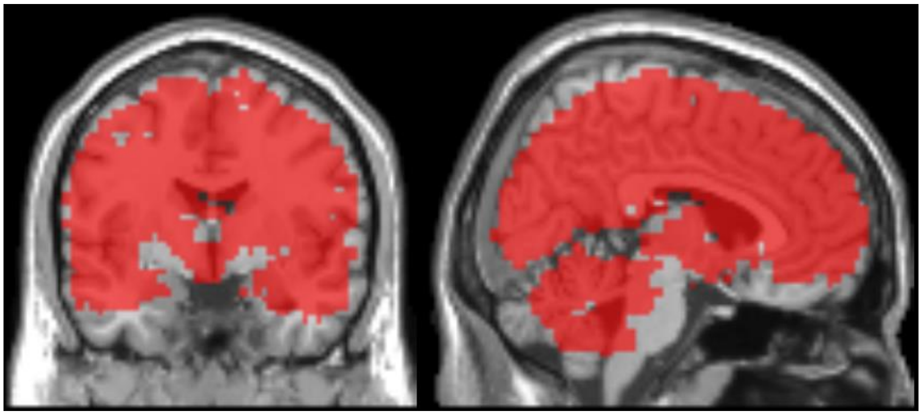

Supplementary Figure 2. Sample-level signal intensity mask. Binary masks were calculated for each subject, at each time point, thresholded at >70% mean signal intensity (Geerligs et al., 2015), computed over all voxels, using ART (www.nitrc.org/projects/artifact_detect/). Then, a sample-level mask was calculated, across all subjects and time points, using logical conjunction. The resulting sample-level mask (red color) is superimposed on the canonical single-subject T1 template part of SPM12, and displayed in coronal (left) and sagittal (right) sections, using MRIcron (https://www.nitrc.org/projects/mricron/); see Methods section for details.
