# Supplementary material for: Aging and Network Properties: Stability Over Time and Links with Learning during Working Memory Training
Source: Front Aging Neurosci. 2018 Jan 4;9:419. doi: 10.3389/fnagi.2017.00419 (PMC5758500; doi:10.3389/fnagi.2017.00419)
Supplement: Supplementary file 3 [file Image3.pdf]

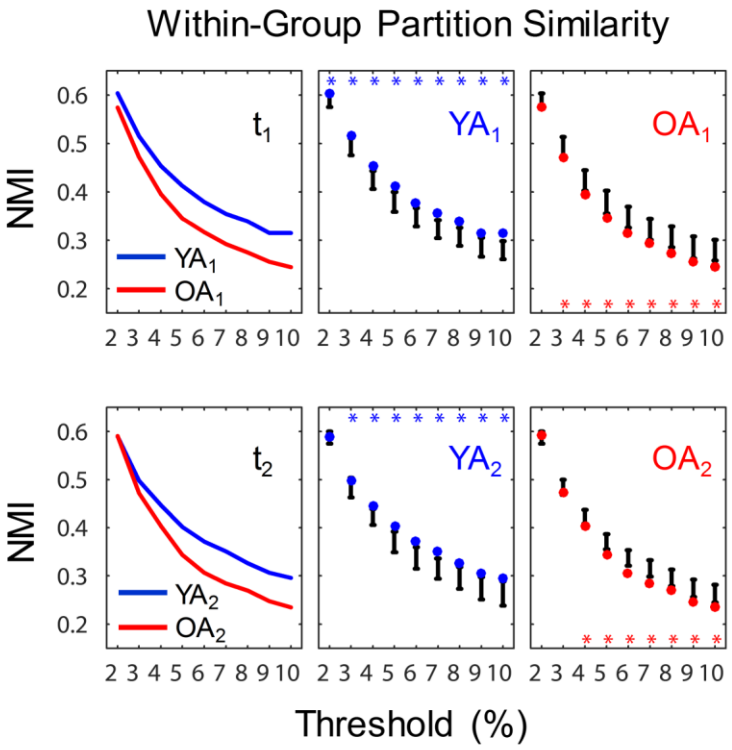

Supplementary Figure 3. Age Differences in Within-Group Partition Similarity. Within-group partition similarity for YA was higher, whereas for OA was lower, than expected based on the permuted data, at both time points, and for most of the threshold range. NMI, normalized mutual information; t_1, time point 1 (top panel); t_2, time point 2 (bottom panel); YA, younger adults (blue color); OA, older adults (red color). Color asterisks indicate $p < 0.05$ for each threshold.
